# Nutritional Epidemiology of Type 2 Diabetes and Depressive Symptoms

**DOI:** 10.2188/jea.JE20130018

**Published:** 2013-07-05

**Authors:** Akiko Nanri

**Affiliations:** Department of Epidemiology and Prevention, Clinical Research Center, National Center for Global Health and Medicine, Tokyo, Japan

**Keywords:** depressive symptoms, diet, Japanese, type 2 diabetes

## Abstract

In Japan, the prevalences of type 2 diabetes and depression are increasing, but evidence linking these diseases to diet is limited. The present study reviewed the association of type 2 diabetes with intakes of rice, fish/seafood, and soy product and isoflavone, and the association of depressive symptoms with folate, vitamin D, and dietary pattern, in the Japanese population. The analysis of type 2 diabetes comprised around 55 000 men and women aged 45 to 75 years who completed a questionnaire for the Japan Public Health Center-based Prospective Study and were free of type 2 diabetes at baseline. The odds ratio of self-reported physician-diagnosed type 2 diabetes during the subsequent 5 years increased with rice intake among women and among physically inactive men but decreased with total fish/seafood intake among men. In addition, risk tended to decrease with soy product and isoflavone intake among overweight and postmenopausal women. Depressive symptoms were assessed using the Center for Epidemiologic Studies Depression Scale. The participants were approximately 530 workers aged 21 to 67 years who participated in a health survey at the time of a periodic health check. A cross-sectional and prospective inverse association between serum folate and depressive symptoms was observed. Serum 25-hydroxyvitamin D level was suggestively associated with decreased prevalence of depressive symptoms in late autumn. In addition, a healthy Japanese pattern—characterized by high intakes of vegetables, fruit, mushrooms, and soy products—was inversely associated with depressive symptoms. The findings suggest that diet has a role in the development of type 2 diabetes and depression in Japanese.

## INTRODUCTION

The prevalence of type 2 diabetes is increasing worldwide.^1^ In Japan the prevalence of diabetes has increased from 6.9 to 8.9 million between 1997 and 2007.^2^ Obesity is a strong determinant of type 2 diabetes but is much less common among Japanese than among Western populations.^3^ Nevertheless, the prevalence of type 2 diabetes in the Japanese population is not much lower than in some Western populations,^4^ a fact that has been attributed to genetic differences between Asian and white populations.^5^ Limited evidence is available regarding the role of environmental factors, particularly dietary factors, that might account for the epidemic of type 2 diabetes among Japanese. White rice, fish/seafood, and soy products are traditionally consumed by Japanese. As compared with brown rice, white rice has less dietary fiber, vitamins, and minerals^6^—which are associated with decreased risk of type 2 diabetes—and has a higher glycemic index.^7^ The results of an ecologic study suggested that fish intake may play a role in the prevention of type 2 diabetes.^8^ Soy products and isoflavone, a major phytoestrogen found in soybeans, improved glucose tolerance and had an antidiabetic effect in animal studies.^9^

Depression is also an important public health issue worldwide.^10^ Persons with severe depression have limited social life due to their symptoms and are at increased risk of suicide.^10^ In Japan, the number of people with depression is increasing,^11^ and the suicide rate is among the highest in the world: approximately 30 000 deaths from suicide have been recorded since 1998.^12^ In particular, death from suicide has been markedly increasing among men aged 40 to 69 years.^12^ Folate is involved in the metabolism of monoamines, such as serotonin in the brain.^13^ Vitamin D receptor and the vitamin B-activating enzyme 1-α-hydroxylase are widely distributed in human brain tissue.^14^ Thus, these nutrients may be related to mood disorders.

Dietary pattern analysis has generated much interest in nutritional epidemiology. Dietary pattern is an integrated variable that combines consumption of several foods or food groups. Because individuals consume food as part of a diet and not as single items or nutrients, these patterns are expected to have a greater impact than any individual item on disease risk.^15^ In addition, dietary pattern analysis can clarify the actual effect of the overall diet.

In this review, the author examines the association of type 2 diabetes with intakes of rice, fish/seafood, and soy/isoflavone, using data from the Japan Public Health Center-based Prospective Study (JPHC study),^16^^–^^18^ and the association of depressive symptoms with folate, vitamin D, and dietary pattern among a Japanese working population (KOM study).^19^^–^^22^

## DIETARY FACTORS AND TYPE 2 DIABETES

### JPHC Study

The JPHC Study was launched in 1990 (cohort I) and 1993 (cohort II).^23^^,^^24^ The participants in cohort I included residents aged 40 to 59 years in 5 Japanese public health center areas. The participants in cohort II included residents aged 40 to 69 years in 6 Japanese public health center areas. A questionnaire survey was conducted at baseline, 5-year follow-up, and 10-year follow-up. Information on medical histories and health-related lifestyle, smoking, drinking, and dietary habits was obtained at each survey. This study was approved by the Institutional Review Board of the National Cancer Center of Japan.

At the baseline, 5-year follow-up (second), and 10-year follow-up (third) surveys, participants completed a self-administered questionnaire on lifestyle and health. In the present analysis, responses to the second survey were used as baseline data because the questionnaire used for the second survey was more comprehensive than the baseline survey in inquiring about food intakes. At the second survey, a food frequency questionnaire (FFQ) was used to assess average intakes of 147 food and beverage items during the previous year.^25^ For most food items, 9 options for frequency of consumption were provided, ranging from almost none to 7 or more times/day. A standard portion size was specified for each food, and respondents were asked to choose their usual portion size from 3 options (ie, less than one-half, standard, or more than 1.5 times). Daily food intake was calculated by multiplying daily frequency of consumption and the standard portion size by its usual portion size. Daily nutrient intake was calculated using the Standard Tables of Food Composition in Japan.^6^ Intake of isoflavones (daidzein and genistein) was calculated based on a food composition table specifically developed to assess isoflavone content in Japanese foods.^26^^,^^27^

Type 2 diabetes that was newly diagnosed during the 5-year period after the second survey was identified by the self-administered questionnaire at the third survey. At the third survey, study participants were asked if they had ever received a diagnosis of diabetes, and if so, when the initial diagnosis was made. Only participants who had received a diagnosis after the second survey (ie, the baseline of the present analysis) were regarded as incident cases. To confirm the validity of self-reported diabetes, we examined a series of medical records from study participants in 3 districts of the study areas and found that 94% of self-reported diabetes cases were confirmed by medical records.^28^

From the study population of the JPHC Study (*n* = 140 420) at baseline, participants who did not respond to the questionnaire survey at the baseline, second, or third surveys or who reported a history of type 2 diabetes or severe disease before follow-up were excluded. After further exclusion of participants with missing information on exposure variables and those who reported extreme total energy intake, a total of around 55 000 participants (25 000 men and 30 000 women) remained for analysis. During the 5-year period, around 1000 participants were newly diagnosed with diabetes.

### Rice and type 2 diabetes^16^

Among women, rice intake was associated with increased incidence of type 2 diabetes after adjustment for covariates, including body mass index (BMI); the multivariable-adjusted odds ratio (OR) for the highest versus lowest quartile category was 1.65 (95% CI, 1.06–2.57; Table 1). Among men, incidence of type 2 diabetes tended to increase with rice intake, though the association was not statistically significant (Table 1). White rice has a high glycemic index,^7^ which is associated with increased risk of type 2 diabetes.^29^ Further, a number of beneficial nutrients including insoluble fiber and magnesium, which are protectively associated with type 2 diabetes, are removed when producing polished white rice.^6^ These facts might explain the finding of a positive association between rice intake and type 2 diabetes. Interestingly, in the present study, subjects with a higher intake of rice tended to have a lower BMI than those with a lower intake, suggesting that rice or a rice-based diet—probably due to its low fat content—may better aid in obesity prevention than a diet with low rice consumption. However, risk of type 2 diabetes was positively associated with rice intake, which suggests that a high rice intake may increase type 2 diabetes risk independent of BMI.

**Table 1.** Multivariable-adjusted odds ratios (95% CI) for type 2 diabetes by quartile of rice and fish intake and quintile of soy product and isoflavone intake

	Quartiles of rice and fish intake					*P* for trend^a^

	Q1 (low)	Q2	Q3	Q4 (high)		
Rice^16^						
Men^b^	1.00 (reference)	1.24 (0.996–1.55)	1.25 (0.93–1.67)	1.19 (0.85–1.68)		0.32
Strenuous physical activity <1 hour/day^c^	1.00 (reference)	1.26 (0.95–1.68)	1.58 (1.09–2.29)	1.36 (0.86–2.17)		0.08
Strenuous physical activity ≥1 hour/day^c^	1.00 (reference)	1.49 (0.93–2.39)	0.83 (0.44–1.55)	1.20 (0.62–2.33)		0.85
Women^b^	1.00 (reference)	1.15 (0.85–1.55)	1.48 (1.08–2.02)	1.65 (1.06–2.57)		0.005
Strenuous physical activity <1 hour/day^c^	1.00 (reference)	1.23 (0.86–1.76)	1.39 (0.95–2.03)	1.55 (0.90–2.66)		0.08
Strenuous physical activity ≥1 hour/day^c^	1.00 (reference)	0.57 (0.24–1.32)	1.27 (0.56–2.87)	1.13 (0.35–3.62)		0.30

Total fish/seafood^17^^,d^						
Men	1.00 (reference)	0.84 (0.67–1.07)	0.80 (0.62–1.03)	0.73 (0.54–1.00)		0.04
Women	1.00 (reference)	1.06 (0.79–1.42)	1.04 (0.75–1.43)	1.01 (0.69–1.49)		0.96
Large fish (men)^17^^,d,e^	1.00 (reference)	0.97 (0.76–1.24)	1.02 (0.79–1.32)	1.06 (0.80–1.41)		0.63
Small and medium-sized fish (men)^17^^,d,e^	1.00 (reference)	0.86 (0.67–1.09)	0.82 (0.63–1.06)	0.68 (0.50–0.92)		0.02
Oily fish (men)^17^^,d,e^	1.00 (reference)	0.94 (0.75–1.19)	0.88 (0.69–1.12)	0.79 (0.59–1.05)		0.10
Lean fish (men)^17^^,d,e^	1.00 (reference)	0.99 (0.78–1.26)	0.95 (0.73–1.23)	1.05 (0.80–1.38)		0.83


	Quintiles of soy product and genistein intake					*P* for trend^a^
	
	Q1 (low)	Q2	Q3	Q4	Q5 (high)	
Soy products^18^^,f^						
Men	1.00 (reference)	1.05 (0.82–1.36)	1.00 (0.76–1.30)	1.00 (0.76–1.32)	1.02 (0.75–1.38)	0.95
Women	1.00 (reference)	0.84 (0.62–1.13)	0.93 (0.69–1.25)	0.77 (0.55–1.06)	0.98 (0.70–1.39)	0.73
BMI <25 kg/m^2^	1.00 (reference)	0.82 (0.53–1.28)	1.05 (0.69–1.61)	0.82 (0.51–1.32)	0.93 (0.55–1.56)	0.80
BMI ≥25 kg/m^2^	1.00 (reference)	0.78 (0.52–1.18)	0.79 (0.52–1.20)	0.62 (0.39–0.99)	0.89 (0.55–1.44)	0.41
Premenopause	1.00 (reference)	1.60 (0.82–3.13)	1.11 (0.53–2.35)	1.01 (0.45–2.27)	1.26 (0.49–3.23)	0.92
Postmenopause	1.00 (reference)	0.75 (0.53–1.07)	0.96 (0.68–1.36)	0.72 (0.50–1.05)	1.01 (0.68–1.50)	0.97
Genistein^18^^,f^						
Men	1.00 (reference)	1.05 (0.82–1.36)	1.16 (0.91–1.51)	1.00 (0.78–1.33)	0.95 (0.77–1.32)	0.71
Women	1.00 (reference)	0.71 (0.52–0.96)	0.88 (0.65–1.19)	0.81 (0.59–1.11)	0.90 (0.63–1.29)	0.77
BMI <25 kg/m^2^	1.00 (reference)	0.69 (0.44–1.09)	0.99 (0.64–1.54)	0.99 (0.63–1.57)	0.73 (0.43–1.27)	0.76
BMI ≥25 kg/m^2^	1.00 (reference)	0.65 (0.43–0.99)	0.74 (0.49–1.14)	0.59 (0.37–0.94)	0.91 (0.56–1.50)	0.57
Premenopause	1.00 (reference)	1.65 (0.82–3.31)	1.29 (0.59–2.82)	1.48 (0.64–3.39)	1.48 (0.56–3.90)	0.54
Postmenopause	1.00 (reference)	0.59 (0.41–0.85)	0.85 (0.60–1.20)	0.73 (0.51–1.05)	0.84 (0.56–1.26)	0.67

Abbreviations: BMI, body mass index; Q, quartile or quintile.

^a^Based on multiple logistic regression analysis with assignment of ordinal numbers to categories of each food or nutrient.

^b^Adjusted for age, study area, BMI, smoking status, alcohol consumption, family history of diabetes mellitus, total physical activity, history of hypertension, occupation, total energy intake, coffee consumption, and intakes of calcium, magnesium, dietary fiber, fruit, vegetables, fish, bread, and noodles.

^c^Adjusted for the all variables in footnote b except total physical activity.

^d^Adjusted for all variables in footnote b except occupation and intakes of fish, bread, and noodles, but including meat and rice intake.

^e^Large fish: salmon, skipjack/tuna, cod/flatfish, and sea bream; small/medium-sized fish: horse mackerel/sardine, saury/mackerel, and eel; oily fish: salmon, horse mackerel/sardine, saury/mackerel, eel, and sea bream; lean fish: skipjack/tuna and cod/flatfish.

^f^Adjusted for all variables in footnote b except occupation, total physical activity, and intakes of fruit, bread, and noodles, but including leisure-time physical activity and green tea consumption.

There was a marginally significant trend association between rice consumption and type 2 diabetes risk among men and women who did not engage in strenuous physical activity at work or during leisure time but not among those who did engage in such activity (Table 1). These results underscore the importance of physical activity in assessing the diet–diabetes risk relation. More specifically, low physical activity may be necessary for rice consumption to affect type 2 diabetes risk.

In a recent meta-analysis of 7 prospective cohorts in 4 reports,^30^ high intake of white rice was significantly associated with increased risk of type 2 diabetes, especially among Asian populations. The pooled relative risk of type 2 diabetes for the highest category of white rice intake was 1.55 (95% CI, 1.20–2.01) as compared with the lowest category among Asian populations, whereas the corresponding value was 1.12 (95% CI, 0.94–1.33) in Western populations. The Japanese rice-centered diet is characterized by low fat intake and has major advantages over the Western diet in terms of contributing to cardiovascular health^31^; however, the same may not be true for preventing type 2 diabetes. Among Japanese who consume rice as a major staple food, dietary strategies that prevent type 2 diabetes without increasing risk of cardiovascular disease might need to be explored.

### Fish and type 2 diabetes^17^

Among men, total fish/seafood intake was inversely associated with type 2 diabetes risk; the multivariable-adjusted OR for type 2 diabetes in the highest versus the lowest quartile of intake was 0.73 (95% CI, 0.54–1.00; Table 1). Additional adjustment for vitamin D and polyunsaturated fatty acid intakes attenuated the association. Among women, total fish/seafood intake was not associated with type 2 diabetes risk. In an analysis by fish size, intake of small/medium-sized fish significantly decreased the risk of type 2 diabetes among men, whereas intake of large fish was not associated with type 2 diabetes (Table 1). Among men, type 2 diabetes risk tended to decrease with oily fish intake but not with lean fish intake (Table 1). Fish, especially oily fish, are a major dietary source of n-3 fatty acids and vitamin D, which were found to be associated with decreased risk of type 2 diabetes.^32^^,^^33^ In the present study, the inverse association between total fish/seafood intake and type 2 diabetes was attenuated after adjustment for vitamin D and polyunsaturated fatty acids. The decreased risk of type 2 diabetes associated with fish intake might be due not only to vitamin D and polyunsaturated fatty acids but also to other components such as fish protein or the synergistic effect of several nutrients.

Some^34^^–^^36^ but not all Western studies have reported an increased risk of type 2 diabetes associated with fish intake. Environmental contaminants, such as dioxins in fish, may increase risk of type 2 diabetes.^37^ However, among the present Japanese population, which had a much higher fish intake than that in Western populations,^2^^,^^38^ the risk of type 2 diabetes was not positively associated with total fish/seafood intake or intake of large fish, which are higher in the food chain and thus likely to have higher concentrations of environmental contaminants.

The inconsistent finding of no association in Western studies and an inverse association in the present study might be partly due to the use of different cooking methods. As compared with raw fish, deep-fried fish has higher concentrations of contaminants^39^ and fewer potential health benefits.^40^ Patel et al^41^ observed an inverse association of type 2 diabetes with intake of non-fried fish (fresh, frozen, or canned) but not fried fish. Among the present participants, grilling (58.9%) and stewing (26.4%) of seafood were much more common than frying (3.9%). High consumption of non-fried fish among Japanese might partly account for the inverse association between fish intake and type 2 diabetes.

The findings from the JPHC Study provide evidence of a protective role of fish intake against type 2 diabetes in Japanese men. However, a recent meta-analysis of 13 cohorts showed that total fish intake and risk of type 2 diabetes were inversely associated among Asian/Australian populations, not associated among European populations, and positively associated in US studies.^42^ Therefore, the association between fish intake and type 2 diabetes warrants further investigation, and the biological mechanisms underlying the inverse association among Asian populations need to be clarified.

### Soy products, isoflavone, and type 2 diabetes^18^

Overall, type 2 diabetes was not associated with intakes of soy products, daidzein, or genistein in either men or women, although somewhat lower ORs were observed among women in the higher intake categories (Table 1). In an analysis stratified by BMI, type 2 diabetes risk in overweight women (BMI ≥25 kg/m^2^) was significantly lower in the fourth versus the lowest quintile of intakes of soy products, daidzein, and genistein (Table 1). Overweight women in the fourth quintile of intake had an approximately 40% lower risk of developing type 2 diabetes than those in the lowest quintile. Such decreases in ORs were not observed in women with a BMI of less than 25 kg/m^2^. Given that obesity induces insulin resistance,^43^ soy products and isoflavones may reduce risk of type 2 diabetes by improving insulin sensitivity.

When the association of soy products with type 2 diabetes was further examined by menopausal status, postmenopausal women in the fourth versus the lowest quintile of soy intake had an approximately 30% lower risk of type 2 diabetes, although the trend association was not clear. In contrast, no such association was observed among premenopausal women. Isoflavones are structurally similar to endogenous estrogens and thus have a weak estrogenic effect.^44^^,^^45^ Estrogen may have a role in glucose homeostasis by modulating expression of genes involved in insulin sensitivity and glucose uptake.^46^ Further, estrogen is a major regulator of adipocyte development and adipocyte number and inhibits lipogenesis by reducing the activity of lipoprotein lipase, an enzyme that regulates lipid uptake by adipocytes.^45^

## DIETARY FACTORS AND DEPRESSIVE SYMPTOMS

### KOM Study

Surveys were conducted in July and November 2006 among employees of 2 municipal offices in northeastern Kyushu, Japan. All full-time workers (*n* = 601) attending a periodic health examination were invited to participate, except those on long sick leave or maternity leave. Participants were asked to fill out survey questionnaires beforehand. The questionnaires were checked for completeness by research staff and, where necessary, answers were clarified by requesting the necessary information from the subjects during the examination. Routinely collected information from the health examination, including anthropometric measurements, biochemical data, and information on medical history, smoking, and alcohol drinking were also obtained. This study was approved by the ethics committee of the National Center for Global Health and Medicine. Written informed consent was obtained from all participants.

Dietary habits during the preceding month were assessed using a validated brief self-administered diet history questionnaire (BDHQ).^47^ Dietary intakes of 58 food and beverage items, energy, and selected nutrients were estimated using an ad hoc computer algorithm for the BDHQ, with reference to the Standard Tables of Food Composition in Japan.^6^ To derive dietary patterns, principal component analysis was performed based on energy-adjusted intakes using a density method of 52 food and beverage items. The factors were rotated by orthogonal transformation (varimax rotation) to maintain uncorrelated factors and ensure greater interpretability. The present study determined 3 factors with eigenvalues, the scree test, and the interpretability of the factors. Dietary patterns were named according to the food items with the highest loadings on each of the 3 factors. The factor scores for each dietary pattern were calculated for each participant by summing intakes of food items weighted by their factor loadings.

Serum folate and 25-hydroxyvitamin D concentrations were measured at an external laboratory (Mitsubishi Chemical Medience, Tokyo, Japan) using a chemiluminescent immunoassay and a competitive protein-binding assay, respectively.

Depressive symptoms were assessed using the Japanese version^48^ of the Center for Epidemiologic Studies Depression (CES-D) scale,^49^ which was included in the lifestyle questionnaire in both surveys. The criterion validity of the CES-D scale has been well established in Western^49^ and Japanese^48^ subjects. Depressive symptoms were defined as present when subjects had a CES-D score of 16 or higher. Another cutoff value (≥19), which may be more suitable for Japanese,^50^ was also used.

Of the 601 eligible workers, 547 (age 21–67 years) participated in the survey (response rate 91%). After exclusion of participants with missing information for any of the analyzed variables, 521 to 530 workers (approximately 310 men and 215 women) remained for each analysis. Of these, approximately 36% had depressive symptoms.

### Serum folate and depressive symptoms^19^**^,^**^22^

Serum folate concentration was inversely associated with prevalence of depressive symptoms among men (*P* for trend = 0.03) but not among women (Table 2). Among men, the multivariable-adjusted ORs for depressive symptoms were approximately 50% to 70% lower in the upper 3 quartiles of serum folate versus the lowest quartile. The inverse association was more evident when the higher cutoff (CES-D scale score of ≥19) was used (Table 2). The results were not materially different after adjustment for homocysteine. Among women, there was no association. Folate is involved in the metabolism of monoamines like serotonin in the brain.^13^ Moreover, 5-methyltetrahydrofolate is required in the synthesis of methionine from homocysteine, which has a neurotoxic effect.^13^ Methionine is a precursor of S-adenosylmethionine, the methyl donor in many methylation reactions in the brain involving neurotransmitters, nucleic acids, proteins, and phospholipids.^13^^,^^51^ S-adenosylmethionine also has antidepressant properties.^13^^,^^51^

**Table 2.** Multivariable-adjusted odds ratios (95% CI) of depressive symptoms by tertile or quartile of serum folate, 25-hydroxyvitamin D, and dietary pattern

	Quartiles of serum folate concentrations				*P* for trend^a^

	Q1 (low)	Q2	Q3	Q4 (high)	
Serum folate (cross-sectional association)^22^					
Men^b^					
CES-D score ≥16	1.00 (reference)	0.53 (0.27–1.03)	0.33 (0.16–0.68)	0.51 (0.25–1.03)	0.03
CES-D score ≥19	1.00 (reference)	0.61 (0.31–1.23)	0.37 (0.17–0.82)	0.35 (0.16–0.75)	0.003
Women^b^					
CES-D score ≥16	1.00 (reference)	0.93 (0.40–2.18)	0.92 (0.41–2.08)	0.91 (0.38–2.16)	0.83
CES-D score ≥19	1.00 (reference)	0.67 (0.26–1.71)	1.03 (0.44–2.42)	0.80 (0.31–2.03)	0.89


	Tertiles of serum folate concentrations				*P* for trend^a^
	
	T1 (low)	T2	T3 (high)		
Serum folate (prospective association)^19^^,c^					
CES-D score ≥16	1.00 (reference)	0.66 (0.29–1.52)	0.40 (0.16–0.99)		0.047
CES-D score ≥19	1.00 (reference)	0.26 (0.08–0.84)	0.27 (0.08–0.86)		0.02


	Quartiles of serum 25-hydroxyvitamin D concentrations				*P* for trend^a^
	
	Q1 (low)	Q2	Q3	Q4 (high)	
Serum 25-hydroxyvitamin D^21^^,d,e^					
Workplace A (survey in July)	1.00 (reference)	0.60 (0.20–1.76)	0.75 (0.27–2.08)	0.70 (0.24–2.05)	0.62
Workplace B (survey in November)	1.00 (reference)	0.84 (0.45–1.58)	0.83 (0.44–1.58)	0.59 (0.30–1.15)	0.14


	Tertiles of dietary pattern score				*P* for trend^a^
	
	T1 (low)	T2	T3 (high)		
Dietary patterns^20^^,e,f^					
Healthy Japanese pattern	1.00 (reference)	0.99 (0.62–1.59)	0.44 (0.25–0.78)		0.006
Animal food pattern	1.00 (reference)	1.47 (0.93–2.32)	0.97 (0.61–1.55)		0.91
Westernized breakfast pattern	1.00 (reference)	1.02 (0.64–1.64)	1.27 (0.77–2.10)		0.34

Abbreviations: CES-D, Center for Epidemiologic Studies Depression; Q, quartile; T, tertile.

^a^Based on multiple logistic regression analysis with assignment of ordinal numbers to categories of each food or nutrient.

^b^Adjusted for age, workplace, job position, marital status, job physical activity, non-job physical activity, smoking status, and alcohol consumption.

^c^Association of serum folate at baseline (in 2006) with prevalence of depressive symptoms at follow-up survey (in 2009); adjusted for all variables in footnote b, plus sex and CES-D scale score at baseline.

^d^Adjusted for all variables in footnote b except workplace, but including sex, BMI, and dietary folate intake.

^e^Depressive symptoms was defined as a CES-D scale score ≥16.

^f^Healthy Japanese pattern: high intakes of vegetables, fruit, soy products, mushrooms, and green tea. Animal food pattern: high intakes of fish/shellfish, meat, processed meat, mayonnaise, and egg. Westernized breakfast pattern: high intakes of bread, confectioneries, milk/yogurt, mayonnaise, and egg and low intakes of rice, alcohol, and fish. Adjusted for all variables in footnote b, plus sex, BMI, history of hypertension, history of diabetes mellitus, and total dietary intake.

Several studies have reported an association between blood folate concentration and depressive symptoms. However, few studies have prospectively examined this association.^52^^–^^54^ Therefore, to further examine the prospective association between folate and depressive symptoms, in 2009 a follow-up survey was conducted at the same municipal offices, 3 years after the baseline survey (in 2006). Of the 547 subjects who participated in the baseline survey, those with depressive symptoms at baseline and nonparticipants in the follow-up survey were excluded. Ultimately, 272 participants remained for analysis. Serum folate concentration at baseline was inversely associated with incidence of depressive symptoms after 3 years; the multivariable-adjusted OR for depressive symptoms associated with the highest versus the lowest tertile of serum folate concentration was 0.40 (95% CI, 0.16–0.99; Table 2). The decreased risk of depressive symptoms associated with higher serum folate concentrations was more evident in men; the multivariable-adjusted ORs (95% CI) for depressive symptoms for the lowest through the highest tertile of serum folate concentration were 1.00 (reference), 0.63 (0.22–1.82), and 0.16 (0.03–0.87) (*P* for trend = 0.03).

The association was then evaluated prospectively, and a decreased incidence of depressive symptoms was observed among participants with higher serum folate concentrations at baseline, which suggests that folate decreases depression risk rather than depression status reduces folate level.

### Serum vitamin D and depressive symptoms^21^

No association was observed between serum 25-hydroxyvitamin D and depressive symptoms. Depressive symptoms and vitamin D concentration vary seasonally. Analysis of the seasonality of the association revealed a differential association. In a November survey of workplace B the prevalence of depressive symptoms tended to decrease with serum 25-hydroxyvitamin D concentration (Table 2). In contrast, a July survey of workplace A showed no such association. Higher prevalence of depressive symptoms during winter, when vitamin D insufficiency is common, has led to the hypothesis that vitamin D is involved in the seasonality of mood.^14^ The present finding of a protective effect of vitamin D on depressive symptoms in late autumn is compatible with this hypothesis. Although we had no information on the seasonality of symptoms, it is reasonable to assume that depressive symptoms in persons who received the survey in late autumn were more likely to be associated with vitamin D insufficiency than those in persons surveyed in summer.

### Dietary patterns and depressive symptoms^20^

The present study identified 3 dietary patterns: healthy Japanese, animal food, and Westernized breakfast. The healthy Japanese dietary pattern is characterized by high intakes of vegetables, fruit, soy products, mushrooms, and green tea and was significantly associated with decreased prevalence of depressive symptoms (Table 2). When subjects with a history of mental disease were excluded, the multivariable-adjusted ORs (95% CI) for depressive symptoms in the lowest through the highest tertile of the dietary pattern were 1.00 (reference), 1.00 (0.62–1.62), and 0.40 (0.22–0.71), respectively (*P* for trend = 0.002). Neither the animal dietary pattern nor the Westernized breakfast pattern was associated with depressive symptoms.

In the present study, as compared with subjects in the lowest tertile of the healthy Japanese dietary pattern score, those in the highest tertile consumed much more folate,^55^ vitamin C,^56^ and vitamin E,^57^^,^^58^ which were found to be associated with decreased risk of depressive symptoms. As mentioned above, folate is associated with depressive symptoms through its role in the metabolism of monoamines in the brain and is a determinant of homocysteine level.^13^ In addition, oxidative stress caused by reactive oxygen species and defective antioxidant defenses may be involved in the pathophysiology of neuropsychiatric disorders.^59^ Therefore, antioxidant vitamins like vitamin C and vitamin E may help decrease the risk of mood disorders. The clear inverse association with the healthy Japanese dietary pattern may be due to the combined effect of these nutrients on mood.

## CONCLUSIONS

In the JPHC Study, rice intake was associated with increased risk of type 2 diabetes among women and physically inactive men. In contrast, total fish/seafood intake and small/medium-sized fish intake were associated with decreased risk of type 2 diabetes among men. In addition, type 2 diabetes risk tended to decrease with increased soy product and isoflavone intake among overweight women and postmenopausal women. These results suggest that the typical Japanese diet affects type 2 diabetes risk. Regarding depressive symptoms, a cross-sectional and prospective inverse association of serum folate with depressive symptoms and a suggestive inverse association of serum vitamin D in seasons with less sunlight were observed. Moreover, a healthy Japanese dietary pattern—characterized by high intakes of vegetables, fruit, mushrooms, and soy products—was associated with decreased prevalence of depressive symptoms. Although mental disorders are mainly associated with psychosocial factors, the present findings suggest that diet also has a role in the development of depression.

## References

[1] Wild S, Roglic G, Green A, Sicree R, King H Global prevalence of diabetes: estimates for the year 2000 and projections for 2030. Diabetes Care. 2004;27:1047–53 10.2337/diacare.27.5.1047 15111519

[2] Kenko Eiyo Joho Kenkyukai. The National Health and Nutrition Survey in Japan, 2007. Tokyo: Daiichi-shuppan; 2010 (in Japanese).

[3] Huxley R, Omari A, Caterson ID. Obesity and diabetes. In: Ekoe JM, Rewers M, Williams R, Zimmet P, editors. The epidemiology of diabetes mellitus. 2nd ed. Chichester: Wiley-Blackwell; 2008.

[4] International Diabetes Federation. IDF Diabetes Atlas 5th ed. Available from: http://www.idf.org/diabetesatlas/5e/Update2012 [accessed: 30 November 2012].

[5] Yazaki Y, Kadowaki T Combating diabetes and obesity in Japan. Nat Med. 2006;12:73–4 10.1038/nm0106-73 16397574

[6] Science and Technology Agency. Standard Tables of Food Composition in Japan. 5th revised and enlarged ed. Tokyo: Printing Bureau of the Ministry of Finance, 2005 (in Japanese).

[7] Foster-Powell K, Holt SH, Brand-Miller JC International table of glycemic index and glycemic load values: 2002. Am J Clin Nutr. 2002;76:5–56 12081815 10.1093/ajcn/76.1.5

[8] Nkondjock A, Receveur O Fish-seafood consumption, obesity, and risk of type 2 diabetes: an ecological study. Diabetes Metab. 2003;29:635–42 10.1016/S1262-3636(07)70080-0 14707894

[9] Mezei O, Banz WJ, Steger RW, Peluso MR, Winters TA, Shay N Soy isoflavones exert antidiabetic and hypolipidemic effects through the PPAR pathways in obese Zucker rats and murine RAW 264.7 cells. J Nutr. 2003;133:1238–43 12730403 10.1093/jn/133.5.1238

[10] Doris A, Ebmeier K, Shajahan P Depressive illness. Lancet. 1999;354:1369–75 10.1016/S0140-6736(99)03121-9 10533878

[11] Ministry of Health, Labor and Welfare. Patinet survey. Available from: http://www.mhlw.go.jp/toukei/saikin/hw/kanja/10syoubyo/suiihyo18.html#05 [accessed: 8 February 2013].

[12] Metropolitan Police Department. Available from: http://www.npa.go.jp/safetylife/seianki/H22jisatsunogaiyou.pdf [accessed: 31 January 2013].

[13] Bottiglieri T Homocysteine and folate metabolism in depression. Prog Neuropsychopharmacol Biol Psychiatry. 2005;29:1103–12 10.1016/j.pnpbp.2005.06.021 16109454

[14] Berk M, Sanders KM, Pasco JA, Jacka FN, Williams LJ, Hayles AL, Vitamin D deficiency may play a role in depression. Med Hypotheses. 2007;69:1316–9 10.1016/j.mehy.2007.04.001 17499448

[15] Hu FB Dietary pattern analysis: a new direction in nutritional epidemiology. Curr Opin Lipidol. 2002;13:3–9 10.1097/00041433-200202000-00002 11790957

[16] Nanri A, Mizoue T, Noda M, Takahashi Y, Kato M, Inoue M, Rice intake and type 2 diabetes in Japanese men and women: the Japan Public Health Center-based Prospective Study. Am J Clin Nutr. 2010;92:1468–77 10.3945/ajcn.2010.29512 20980490

[17] Nanri A, Mizoue T, Noda M, Takahashi Y, Matsushita Y, Poudel-Tandukar K, Fish intake and type 2 diabetes in Japanese men and women: the Japan Public Health Center-based Prospective Study. Am J Clin Nutr. 2011;94:884–91 10.3945/ajcn.111.012252 21775559

[18] Nanri A, Mizoue T, Takahashi Y, Kirii K, Inoue M, Noda M, Soy product and isoflavone intakes are associated with a lower risk of type 2 diabetes in overweight Japanese women. J Nutr. 2010;140:580–6 10.3945/jn.109.116020 20053935

[19] Nanri A, Hayabuchi H, Ohta M, Sato M, Mishima N, Mizoue T Serum folate and depressive symptoms among Japanese men and women: a cross-sectional and prospective study. Psychiatry Res. 2012;200:349–53 10.1016/j.psychres.2012.04.040 22682152

[20] Nanri A, Kimura Y, Matsushita Y, Ohta M, Sato M, Mishima N, Dietary patterns and depressive symptoms among Japanese men and women. Eur J Clin Nutr. 2010;64:832–9 10.1038/ejcn.2010.86 20485303

[21] Nanri A, Mizoue T, Matsushita Y, Poudel-Tandukar K, Sato M, Ohta M, Association between serum 25-hydroxyvitamin D and depressive symptoms in Japanese: analysis by survey season. Eur J Clin Nutr. 2009;63:1444–7 10.1038/ejcn.2009.96 19690578

[22] Nanri A, Mizoue T, Matsushita Y, Sasaki S, Ohta M, Sato M, Serum folate and homocysteine and depressive symptoms among Japanese men and women. Eur J Clin Nutr. 2010;64:289–96 10.1038/ejcn.2009.143 20087384

[23] Tsugane S, Sobue T Baseline survey of JPHC study—design and participation rate. Japan Public Health Center-based Prospective Study on Cancer and Cardiovascular Diseases. J Epidemiol. 2001;11(6 Suppl):S24–9 10.2188/jea.11.6sup_24 11763136 PMC11858361

[24] Watanabe S, Tsugane S, Sobue T, Konishi M, Baba S Study design and organization of the JPHC study. Japan Public Health Center-based Prospective Study on Cancer and Cardiovascular Diseases. J Epidemiol. 2001;11(6 Suppl):S3–7 10.2188/jea.11.6sup_3 11763137 PMC11858366

[25] Sasaki S, Kobayashi M, Ishihara J, Tsugane S; JPHC Self-administered food frequency questionnaire used in the 5-year follow-up survey of the JPHC Study: questionnaire structure, computation algorithms, and area-based mean intake. J Epidemiol. 2003;13(1 Suppl):S13–22 10.2188/jea.13.1sup_13 12701629 PMC9767697

[26] Arai Y, Watanabe S, Kimira M, Shimoi K, Mochizuki R, Kinae N Dietary intakes of flavonols, flavones and isoflavones by Japanese women and the inverse correlation between quercetin intake and plasma LDL cholesterol concentration. J Nutr. 2000;130:2243–50 10958819 10.1093/jn/130.9.2243

[27] Kimira M, Arai Y, Shimoi K, Watanabe S Japanese intake of flavonoids and isoflavonoids from foods. J Epidemiol. 1998;8:168–75 10.2188/jea.8.168 9782673

[28] Kato M, Noda M, Inoue M, Kadowaki T, Tsugane S; JPHC Study Group Psychological factors, coffee and risk of diabetes mellitus among middle-aged Japanese: a population-based prospective study in the JPHC study cohort. Endocr J. 2009;56:459–68 10.1507/endocrj.K09E-003 19270421

[29] Barclay AW, Petocz P, McMillan-Price J, Flood VM, Prvan T, Mitchell P, Glycemic index, glycemic load, and chronic disease risk—a meta-analysis of observational studies. Am J Clin Nutr. 2008;87:627–37 18326601 10.1093/ajcn/87.3.627

[30] Hu EA, Pan A, Malik V, Sun Q White rice consumption and risk of type 2 diabetes: meta-analysis and systematic review. BMJ. 2012;344:e1454 10.1136/bmj.e1454 22422870 PMC3307808

[31] Willett WC Diet and health: what should we eat? Science. 1994;264:532–7 10.1126/science.8160011 8160011

[32] Pittas AG, Lau J, Hu FB, Dawson-Hughes B The role of vitamin D and calcium in type 2 diabetes. A systematic review and meta-analysis. J Clin Endocrinol Metab. 2007;92:2017–29 10.1210/jc.2007-0298 17389701 PMC2085234

[33] Salmerón J, Hu FB, Manson JE, Stampfer MJ, Colditz GA, Rimm EB, Dietary fat intake and risk of type 2 diabetes in women. Am J Clin Nutr. 2001;73:1019–26 11382654 10.1093/ajcn/73.6.1019

[34] Djoussé L, Gaziano JM, Buring JE, Lee IM Dietary omega-3 fatty acids and fish consumption and risk of type 2 diabetes. Am J Clin Nutr. 2011;93:143–50 10.3945/ajcn.110.005603 20980491 PMC3001602

[35] Kaushik M, Mozaffarian D, Spiegelman D, Manson JE, Willett WC, Hu FB Long-chain omega-3 fatty acids, fish intake, and the risk of type 2 diabetes mellitus. Am J Clin Nutr. 2009;90:613–20 10.3945/ajcn.2008.27424 19625683 PMC2728645

[36] van Woudenbergh GJ, van Ballegooijen AJ, Kuijsten A, Sijbrands EJ, van Rooij FJ, Geleijnse JM, Eating fish and risk of type 2 diabetes: A population-based, prospective follow-up study. Diabetes Care. 2009;32:2021–6 10.2337/dc09-1042 19675200 PMC2768220

[37] Rignell-Hydbom A, Lidfeldt J, Kiviranta H, Rantakokko P, Samsioe G, Agardh CD, Exposure to p,p′-DDE: a risk factor for type 2 diabetes. PLoS One. 2009;4:e7503 10.1371/journal.pone.0007503 19838294 PMC2759028

[38] Wang Y, Beydoun MA, Caballero B, Gary TL, Lawrence R Trends and correlates in meat consumption patterns in the US adult population. Public Health Nutr. 2010;13:1333–45 10.1017/S1368980010000224 20188005 PMC2916052

[39] Burger J, Dixon C, Boring CS, Gochfeld M Effect of deep-frying fish on risk from mercury. J Toxicol Environ Health A. 2003;66:817–28 10.1080/15287390306382 12746129

[40] Candela M, Astiasarán I, Bello J Deep-fat frying modifies high-fat fish lipid fraction. J Agric Food Chem. 1998;46:2793–6 10.1021/jf9709616

[41] Patel PS, Sharp SJ, Luben RN, Khaw KT, Bingham SA, Wareham NJ, Association between type of dietary fish and seafood intake and the risk of incident type 2 diabetes: the European prospective investigation of cancer (EPIC)-Norfolk cohort study. Diabetes Care. 2009;32:1857–63 10.2337/dc09-0116 19592633 PMC2752921

[42] Wallin A, Di Giuseppe D, Orsini N, Patel PS, Forouhi NG, Wolk A Fish consumption, dietary long-chain n-3 fatty acids, and risk of type 2 diabetes: systematic review and meta-analysis of prospective studies. Diabetes Care. 2012;35:918–29 10.2337/dc11-1631 22442397 PMC3308304

[43] Kahn BB, Flier JS Obesity and insulin resistance. J Clin Invest. 2000;106:473–81 10.1172/JCI10842 10953022 PMC380258

[44] Bhathena SJ, Velasquez MT Beneficial role of dietary phytoestrogens in obesity and diabetes. Am J Clin Nutr. 2002;76:1191–201 12450882 10.1093/ajcn/76.6.1191

[45] Ørgaard A, Jensen L The effects of soy isoflavones on obesity. Exp Biol Med (Maywood). 2008;233:1066–80 10.3181/0712-MR-347 18535167

[46] Barros RP, Machado UF, Gustafsson JA Estrogen receptors: new players in diabetes mellitus. Trends Mol Med. 2006;12:425–31 10.1016/j.molmed.2006.07.004 16890492

[47] Kobayashi S, Murakami K, Sasaki S, Okubo H, Hirota N, Notsu A, Comparison of relative validity of food group intakes estimated by comprehensive and brief-type self-administered diet history questionnaires against 16 d dietary records in Japanese adults. Public Health Nutr. 2011;14:1200–11 10.1017/S1368980011000504 21477414

[48] Shima S, Shikano T, Kitamura T, Asai M New self-rating scale for Depression. Jpn J Clin Psychiatry. 1985;27:717–23 (in Japanese)

[49] Radloff LS The CES-D scale: a self-report depression scale for research in the general population. Appl Psychol Meas. 1977;1:385–401 10.1177/014662167700100306

[50] Wada K, Tanaka K, Theriault G, Satoh T, Mimura M, Miyaoka H, Validity of the Center for Epidemiologic Studies Depression Scale as a screening instrument of major depressive disorder among Japanese workers. Am J Ind Med. 2007;50:8–12 10.1002/ajim.20403 17096372

[51] Reynolds E Vitamin B12, folic acid, and the nervous system. Lancet Neurol. 2006;5:949–60 10.1016/S1474-4422(06)70598-1 17052662

[52] Eussen SJ, Ferry M, Hininger I, Haller J, Matthys C, Dirren H Five year changes in mental health and associations with vitamin B12/folate status of elderly Europeans. J Nutr Health Aging. 2002;6:43–50 11813081

[53] Kendrick T, Dunn N, Robinson S, Oestmann A, Godfrey K, Cooper C, A longitudinal study of blood folate levels and depressive symptoms among young women in the Southampton Women’s Survey. J Epidemiol Community Health. 2008;62:966–72 10.1136/jech.2007.069765 18854500 PMC4558940

[54] Kim JM, Stewart R, Kim SW, Yang SJ, Shin IS, Yoon JS Predictive value of folate, vitamin B12 and homocysteine levels in late-life depression. Br J Psychiatry. 2008;192:268–74 10.1192/bjp.bp.107.039511 18378986

[55] Gilbody S, Lightfoot T, Sheldon T Is low folate a risk factor for depression? A meta-analysis and exploration of heterogeneity. J Epidemiol Community Health. 2007;61:631–7 10.1136/jech.2006.050385 17568057 PMC2465760

[56] Smith AP, Clark RE, Nutt DJ, Haller J, Hayward SG, Perry K Vitamin C, mood and cgnitive fuctioning in the elderly. Nutr Neurosci. 1999;2:249–56 10.1080/1028415X.1999.11747281 27415576

[57] Shibata H, Kumagai S, Watanabe S, Suzuki T Relationship of serum cholesterols and vitamin E to depressive status in the elderly. J Epidemiol. 1999;9:261–7 10.2188/jea.9.261 10510584

[58] Tiemeier H, Hofman A, Kiliaan AJ, Meijer J, Breteler MM Vitamin E and depressive symptoms are not related. The Rotterdam Study. J Affect Disord. 2002;72:79–83 10.1016/S0165-0327(01)00427-X 12204320

[59] Bilici M, Efe H, Köroğlu MA, Uydu HA, Bekaroğlu M, Değer O Antioxidative enzyme activities and lipid peroxidation in major depression: alterations by antidepressant treatments. J Affect Disord. 2001;64:43–51 10.1016/S0165-0327(00)00199-3 11292519

